# At-Home Sleep Electroencephalography Assessment in Young and Older Adults Using a Novel Wireless Soft Electronics Sleep Monitoring System: Experimental Study

**DOI:** 10.2196/80286

**Published:** 2026-05-04

**Authors:** Hyeonseok Kim, Simran Saha, Aiden Wachnin, Seunghyeb Ban, Yoon Jae Lee, Youngjin Kwon, Jaeho Lee, Isha Chhabra, Sahana Ram, Chuu Nyan, Lynn Marie Trotti, Woon-Hong Yeo, Audrey Duarte

**Affiliations:** 1 George W. Woodruff School of Mechanical Engineering, Wearable Intelligent Systems and Healthcare Center (WISH Center), Georgia Institute of Technology, Atlanta, GA, United States; 2 Department of Psychology, The University of Texas at Austin, 108 East Dean Keeton Street NW, Austin, TX, 78712, United States, 1 5122324643; 3 Department of Neurology, Emory Sleep Center, Emory University, Atlanta, GA, United States

**Keywords:** aging, sleep, sleep patterns, wearables, electroencephalography, naturalistic, validation

## Abstract

**Background:**

Sleep quality declines with age and is a known contributor to multiple chronic health conditions, including Alzheimer disease. Emerging evidence suggests that certain electroencephalography (EEG) neural signatures measured during sleep may be predictive of cognitive decline in older adults. Sleep EEG signals are traditionally measured using bulky, rigid, and uncomfortable equipment in an unfamiliar laboratory setting, which can negatively impact sleep signals. Due to these limitations, sleep EEG data acquisition is typically limited to a single night.

**Objective:**

This study aimed to validate our recently developed portable, skin-like EEG monitoring patch for 7 nights in the home environment in a pilot sample of young and older adults by evaluating usability and acceptance, and replicating age-related differences in sleep architecture observed in the polysomnography literature.

**Methods:**

Eighteen young adults and 18 cognitively unimpaired older adults without sleep disorders were enrolled (data from 11 young adults and 12 older adults were included in the analyses) in a 7-night study during which they wore novel, gel-free, wireless, ultrathin, skin-conforming, sleep monitoring, fabric-based patches. These patches were self-applied to the forehead and face for optimal usability and comfort. The patches incorporate laser-cut mesh electrodes with low-profile electronics (including a rechargeable battery and amplifier) and transmit EEG signals to a participant-controlled, Bluetooth-enabled, tablet-based data acquisition app. An automated algorithm was used to stage sleep and assess microarchitecture features from the EEG commonly impacted for each participant. Averages across nights were computed for these sleep features for each participant.

**Results:**

Young and older adults reported that the sleep patch was easy to use and comfortable to wear. There was no loss of signal power over 7 nights of wear across participants (retained-data signal-to-noise ratio over the 7-d period: young adult, mean 20.69, SD 12.78, maximum 52.13, minimum 5.19; older adult, mean 22.10, SD 9.39, maximum 49.96, minimum 13.79). Most datasets not retained were lost due to poor reference electrode adhesion on the nose (75/101, 74% of lost datasets in young adults and 57/88, 65% in older adults). Trained sleep technologists verified that the retained datasets were of sufficient quality to be scored without difficulty. Expected age-group differences in sleep features were observed, including age-related reductions in stage N3 sleep (young adult, mean 18.55, SD 6.70; older adult, mean 10.40, SD 6.43; Mann-Whitney *U*=42.0; *P*=.01) and reduced sleep spindle density (young adult, mean 2.92, SD 2.24; older adult, mean 0.94, SD 1.33; Mann-Whitney *U*=45.0; *P*=.006).

**Conclusions:**

This study demonstrates that our novel, comfortable, wearable patch can reliably measure physiological sleep data over multiple nights at home in adults across the lifespan, thereby making multinight sleep assessment in cognitive aging studies and clinical research more accessible than traditional polysomnography. In future studies, the small, lightweight system, which is highly scalable, can be shipped inexpensively to participants’ homes, making this technology and research accessible to individuals who may have difficulty traveling or who are hesitant to travel to a laboratory or clinic.

## Introduction

Although sleep complaints are common across adult age groups, poor sleep is particularly prevalent with increasing age. Large population-based studies show that nearly half of adults older than 65 years report experiencing sleep disturbances each week, including trouble falling asleep and staying asleep [1]. Importantly, acute sleep disruptions, including prior episodes of insomnia, can increase the subsequent risk of chronic insomnia [2], the prevalence of which increases dramatically in older age [3]. Sleep serves many health-promoting functions, including tissue restoration and the clearance of metabolites and neurotoxins [4]. Consequently, poor sleep is associated with an increased risk and continuation of numerous diseases, including diabetes, hypertension, and Alzheimer disease (AD) [5]. Numerous studies have identified an important role for sleep in cognition, particularly memory [6]. As sleep is potentially modifiable, there is a critical need to identify the aspects of disordered sleep that may forecast chronic problems, including cognitive decline and AD, thereby aiding in the development of targeted interventions.

Laboratory-based polysomnography is the “gold standard” method for assessing sleep. It involves recording electroencephalography (EEG) and electromyography (EMG) from gel-filled electrodes placed, most typically, over the face and scalp to monitor neural oscillations and muscle activity, respectively, which are characteristic of different sleep stages. These stages include rapid eye movement (REM) sleep, characterized by low-amplitude, mixed-frequency oscillations and bursts of eye movements, and non-REM (NREM) sleep, comprising stages N1 to N3. NREM stage N3 is characterized by high-amplitude slow-wave activity (SWA) in the delta frequency range (0.5‐4 Hz), while stage N2 is primarily marked by oscillatory bursts of activity in the 10 to 16 Hz range. The relative time spent in sleep stages (macroarchitecture) and the spectral power of these sleep signals (microarchitecture) vary substantially between individuals and predict individual differences in cognitive performance [5,7]. Laboratory-based polysomnography can also include respiratory and electrocardiography signals for measuring and diagnosing sleep-disordered breathing.

There are several limitations to laboratory-based polysomnography. First, participants are required to sleep in laboratories rather than their homes, which limits ecological validity and adversely impacts sleep. Second, current approaches are expensive, with substantial costs for polysomnography equipment and personnel to monitor participants, ultimately creating barriers to accessing care. Third, the polysomnography apparatus is bulky, hardwired, and uncomfortable, with messy electroconductive gels that need to be reapplied due to evaporation [8]. However, dry, gel-free electrode systems typically provide worse signal quality [9]. Due to these limitations, laboratory-based polysomnography research studies tend to have small sample sizes, and data are typically limited to a single night. Sampling a single night only makes it difficult to determine whether poor sleep quality is a state or trait feature. Specifically, the first-night effect is a well-known confound of single-night polysomnography studies, characterized by less total sleep time, more awakenings, and lower sleep efficiency compared to subsequent polysomnography nights due to acclimation [10]. Furthermore, as night-to-night sleep variability is associated with poorer cognitive performance and health [11], it is essential to sample multiple nights of sleep. Mobile polysomnography systems overcome some of these limitations, namely the ability to monitor sleep from home. While several mobile polysomnography systems enable home-based sleep recordings, some implementations may still require auxiliary components beyond the sensing electrodes themselves. Form factor and user experience, therefore, vary across mobile polysomnography platforms.

Compact, low-cost, mobile EEG systems with a reduced number of electrodes for placement on nonhairy skin have been developed to overcome the limitations of laboratory and home-based polysomnography [12-15]. These systems offer short setup times and are more practical for self-application at home, using a range of electrode types, including dry and gel-based electrodes, depending on design trade-offs and intended use, making them amenable to multinight use. For example, wireless headbands have forehead-only dry electrodes, and some have been validated for home use [16,17]. However, existing systems vary in their electronic and structural designs, with trade-offs related to form factor, user comfort, and suitability for prolonged wear and self-application, and may differ in the extent to which additional electrodes (eg, for eye or chin movements) are incorporated for sleep scoring [16,18]. Other low-profile EEG systems employing forehead EEG electrode sets combined with chin EMG electrodes have primarily been evaluated in single-night, attended laboratory polysomnography studies as part of validation protocols [19]. Alternative small form factor devices placed in or around the ear vary in their design and application; some systems require individual customization or prioritize applications such as drowsiness detection, while others may differ in their validated performance for full sleep staging [20-22].

Our team has recently demonstrated soft substrate, ultrathin wearable systems using epidermal electronics [23]. We have developed and validated a gel-free, fully portable, and highly skin-conforming wireless dry electrode at-home sleep monitoring system that integrates soft and functional materials with electronics for a comfortable yet reliable wearable system [24]. A key advantage of our sleep patch lies in its modular design. It includes a reusable all-in-one circuit system with an integrated power package and a biocompatible electrode embedded in breathable fabric for single-participant use. While the electrodes are disposable, the costly circuit components can be recycled to create new patches for future users, reducing fabrication costs and environmental impact. The soft, elastic fabric and silicone elastomer in which the dry electrodes are embedded provide strain distribution to avoid damage during device assembly, handling, and multiple uses [25]. We recently tested our sleep patch system against a traditional polysomnography system over 1 night in our sleep clinic in middle-aged and older participants and showed high manual scoring agreement between these systems (82%, *k*=0.74 across all participants), on par with the highest levels of agreement for other wearable sleep systems in the literature [24]. We further showed in our prior study that this system has robust signal-to-noise ratio (SNR) performance for at least 2 weeks of continuous use in a controlled laboratory setting.

**Figure 1.**
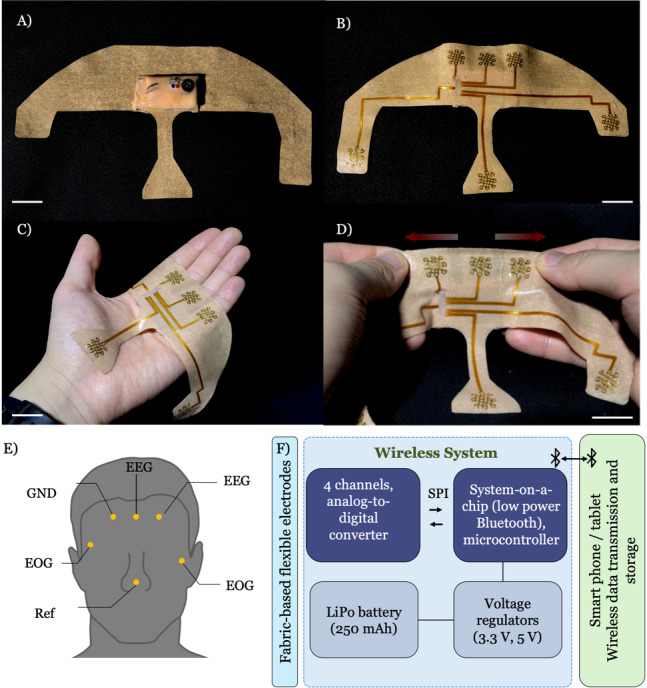
Overview of the portable, wireless, skin-conforming sleep monitoring system. (A) Top view of the sleep monitoring system, which consists of a fabric-based patch with a soft, polymer-encapsulated wireless module. (B) Bottom view of the system, showing the detailed electrode configuration. (C) The sleep monitoring system held in hand, demonstrating its excellent flexibility. (D) The stretchable sleep patch ensures highly skin-conforming contact for stable and comfortable wear during daily use. (E) Measurement locations of the electrodes in the sleep patch for precise electroencephalography and electrooculography monitoring. (F) Schematic diagram illustrating the system operation flow. SPI is a communication protocol that allows digitized bidirectional communication between the processor and ADC (scale bars in A, B, C, D: 2 cm). EEG: electroencephalography; SPI: Serial Peripheral Interface.

In this pilot study, we aim to test the acceptance and feasibility of our sleep monitoring patch prototype (Figure 1) for multinight home sleep EEG recording in young and cognitively unimpaired older adults without sleep disorders. To our knowledge, this is the first assessment of a forehead soft electronics system for home monitoring of sleep EEG. Due to the laser-based manufacturing of electrodes and recyclable circuit components, our system can be inexpensively produced relative to commercially available systems that lack these features, making it highly scalable for shipping to large numbers of participants for future cognitive and clinical studies. This study aims to lay the groundwork for these future studies. Here, we define acceptance as the ability of young and older adults to self-apply the system at home for multiple nights and to endorse its ease of use and comfort during sleep. We define feasibility according to (1) the number of full nights of EEG data recorded for both age groups; (2) the stability of SNR of EEG signals over the multiple nights of use; (3) the ability of trained sleep technologists to verify that the recorded data could be manually scored with little difficulty; and (4) evidence of age group differences in macroarchitecture and microarchitecture features most commonly observed in the literature [26,27]. Specifically, age-associated attenuation of the relative percentage of NREM slow-wave sleep (SWS; N3), REM, and reduced density of slow oscillations (SOs) and spindles during NREM [28,29]. Collectively, these findings would support the reliability, participant acceptance, and feasibility of wireless, wearable EEG devices for sleep monitoring over multiple nights in adults across the lifespan.

## Methods

### Participants

Eighteen young adults and 18 cognitively unimpaired older adults were recruited between March 2023 and January 2025. All participants were recruited from the University of Texas at Austin and the surrounding Austin community using mailings, flyer distribution, and phone calls. Interested individuals completed an initial REDCap (Research Electronic Data Capture) intake form for eligibility screening before trained research staff scheduled enrollment by phone. Participants were enrolled if they self-reported that they were fluent in English, had normal or corrected-to-normal vision, had no history of sleep disorders, and were free from untreated depression or anxiety, psychiatric or neurological disorders, or diabetes. Each participant had the opportunity to use the sleep monitoring system for up to 7 contiguous nights. Of the 18 young adults and 18 older adults recruited, 7 were excluded from the young group and 6 from the older group for failing to have at least 1 full night of usable EEG data. Thus, the analytic sample for sleep macrostructure and microstructure analysis included the remaining 11 young adults (8 women, aged 18‐28 y; mean education 15.1, SD 2.7 y; 4 Non-Hispanic Asian, 1 Hispanic Black, 4 Hispanic White, 2 Non-Hispanic White) and 12 older adults (6 women, aged 64‐79 y; mean education 17, SD 3 y; 1 Non-Hispanic Asian, 1 Non-Hispanic Black, 2 Hispanic White, 7 Non-Hispanic White, 1 did not report). Reasons for data loss are described in Table 1. Older adults completed the California Verbal Learning Test 3 for long-term verbal memory [30] and the Digit Span (Wechsler Adult Intelligence Scale-Fourth Edition) for executive functioning [31] to screen for clinically significant cognitive impairment. To this end, participants scoring 2 SDs below the across-participant mean on these tests would be excluded. No participants were excluded based on their cognitive assessments.

**Table 1.** Sleep electroencephalography data retention.

Panels and measures	Young adults	Older adults
Panel A: Participants with 0 usable nights (young adults: n=7; older adults: n=6), n/N (%)		
Poor reference electrode adhesion	38/49 (78)	26/42 (62)
Not worn and/or not connected	11/49 (22)	16/42 (38)
Bluetooth issues	0/49 (0)	0/42 (0)
Panel B: Participants with 1 or more usable nights (young adults: n=11; older adults: n=12)		
Usable nights (of nights with device use^a^), n/N (%)	25/63 (39.7)	38/70 (54.3)
Not worn/connected (of scheduled nights), n/N (%)	14/77 (18.2)	16.7 (14/84)
Poor reference electrode adhesion (of scheduled nights), n/N (%)	37/77 (48.1)	31/84 (36.9)
Bluetooth issues (of scheduled nights), n/N (%)	1/77 (1.3)	1/84 (1.2)
Signal-to-noise ratio (dB) over the 7-d period for retained data, mean (maximum, minimum)	20.69 (52.13, 5.19)	22.10 (49.96, 13.79)

a “Nights with device use” refers to nights during which the participant wore the patch and successfully initiated recording (ie, the patch was worn and connected to the tablet app). “Usable nights” are a subset of nights with device use that met data-quality criteria for analysis.

### Sleep-Monitoring Patch System

Full details of device fabrication, mechanical reliability, and reusability testing can be found in Kown et al [24]. In the previous version of this system, which was validated in a controlled laboratory setting, an additional chin patch was used for monitoring EMG with a separate reference electrode from the one used to monitor sleep EEG in the forehead patch. To simplify the system for participants applying it at home, we excluded the chin patch. This forehead system is consistent with the forehead-only systems used in most wearable sleep monitoring devices. Our team has developed a portable, wireless, skin-conforming sleep monitoring system that overcomes the limitations of existing home polysomnography and wearable sleep monitoring devices. A composite of elastic fabric and ultrasoft silicone elastomer provides strain distribution, omnidirectional elasticity, strong yet nonsticky adhesion to the skin, and a substrate for the integration of gold, laser-cut mesh electrodes, and copper interconnectors linking the electrodes with electronics (rechargeable battery, amplifier, Bluetooth microcontroller). The electrodes and the integrated circuit are encapsulated on the silicone side of the device, while the circuit—including a Bluetooth wireless module and a rechargeable 150-mAh Li-polymer battery with an average battery life of approximately 10 hours based on repeated laboratory measurements—is located on the fabric side of the device. The fabric provides a dry surface that allows for easy handling by the wearer while simultaneously protecting the electronics against mechanical deformations during wear and handling. The system requires only 1 unobtrusive patch placed on the forehead, with a reference electrode positioned on the nasal bridge to enable single-patch self-application. The patch contains 6 embedded electrodes: 3 electrodes embedded in the forehead region (2 for measuring EEG and 1 to serve as the ground); 1 embedded in the patch placed below the left eye and 1 above the right eye for measuring electrooculography (EOG); and 1 in the patch on the bridge of the nose to serve as the reference. Data were collected at 250 Hz, and real-time signals were transmitted via Bluetooth to a nearby mobile device or tablet. Participants can easily charge the patches, clean them with alcohol spray, and upload data to the provided tablet.

### The Android App

**Figure 2.**
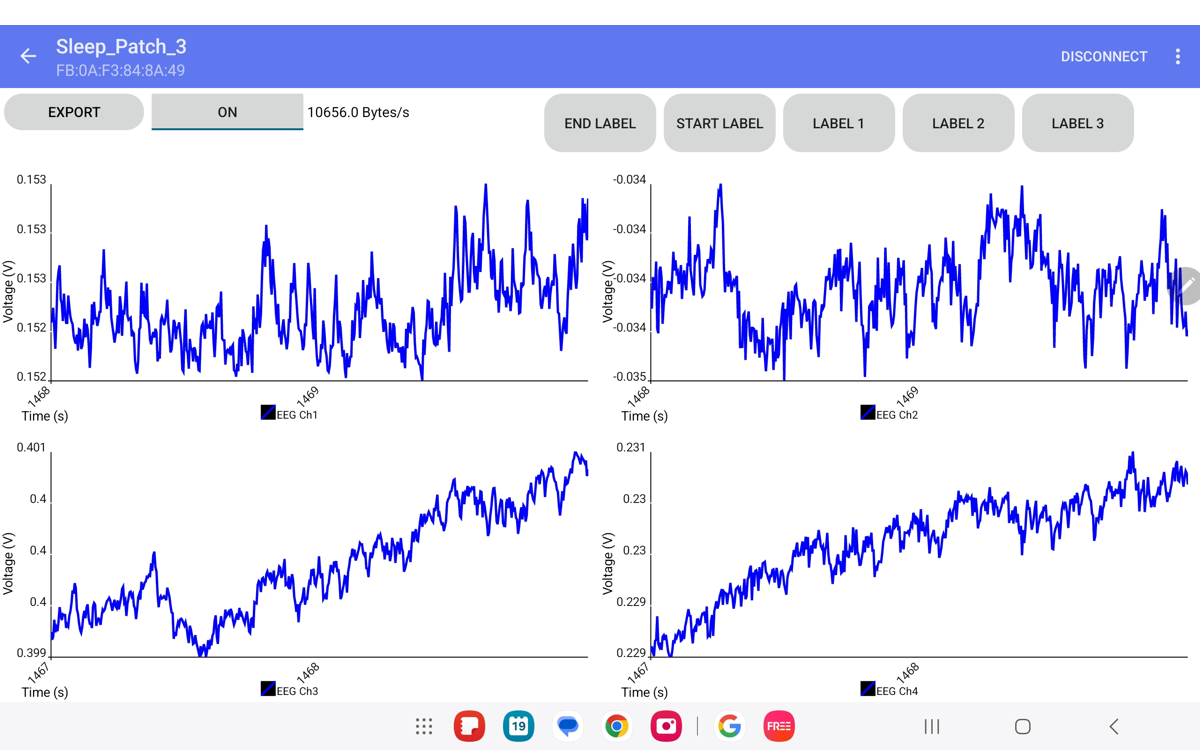
Screen image of the Android app for sleep patch data collection. The electrodes shown (visible to participants) correspond to 2 electroencephalography (EEG) channels (EEG Ch1 and EEG Ch2) and 2 electrooculography channels, which are displayed in the interface as EEG Ch3 and EEG Ch4. Interface label buttons were visible to participants but not functional, as they were used for marking events by researchers during analysis.

An Android app was developed in Kotlin to manage continuous EEG data streaming, time alignment, user-defined event labeling, and local file storage. The app uses the native Bluetooth Low Energy application programming interface to discover and connect to the microcontroller device based on its Universally Unique Identifier and subscribes to characteristic notifications for EEG data packets. In the current implementation, the app supports connection to a single device at a time. Upon receiving each packet, the app parses the raw hexadecimal data into floating buffers in memory to allow real-time visualization of waveforms and concurrent storage on-device. To accommodate 8-hour or longer sleep recordings, data are stored in a structured large CSV format. A periodic file rotation scheme for every 4 buffer sets (<0.05 s) is implemented to reduce the risk of file corruption during battery depletion or app shutdown. An image of the app interface with EEG signals is shown in Figure 2.

### Procedure

**Figure 3.**
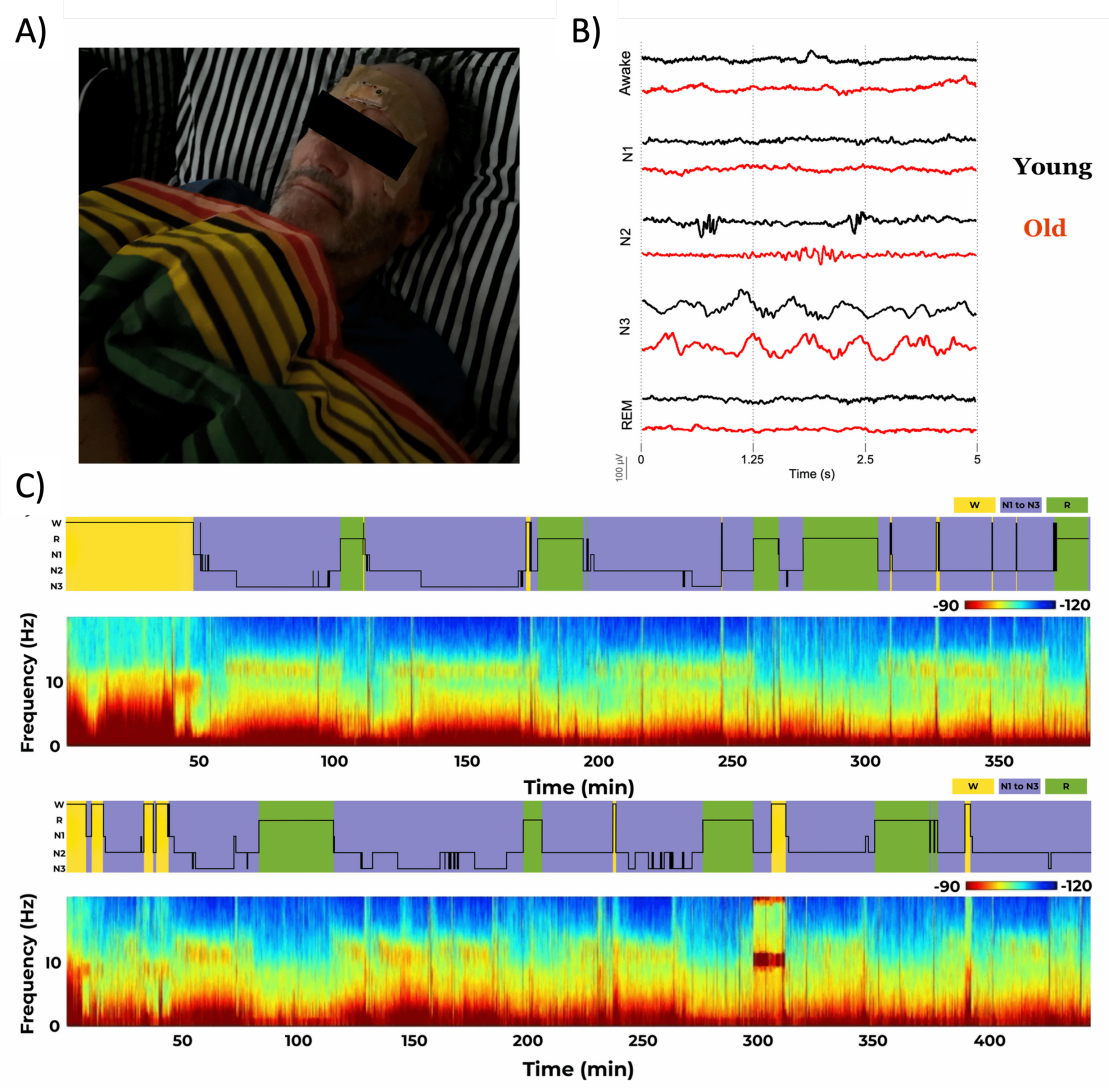
(A) One of our participants wearing the sleep patch at home. (B) Five seconds of electroencephalography data from the patch for each sleep stage for one representative young adult and one older adult. (C) Hypnograms and associated spectrograms for an entire night for one representative young adult and one older adult. REM: rapid eye movement.

Participants were enrolled in a 7-day study that included 3 laboratory visits. They were asked to wear a sleep-monitoring patch for 7 nights (see Figure 3A). Collecting data over seven nights helps protect against potential data loss due to participant errors (eg, failure to charge or apply patches correctly) or device malfunctions. At the first laboratory visit, participants were provided with a sleep-monitoring patch, a Samsung tablet, a Philips Actiwatch 2 accelerometer, a sleep log, and an instruction packet for reference in case they forgot any of the procedures explained during the visit. All data measured by the patch were transmitted via Bluetooth and stored on the Samsung tablet. Participants were instructed to wear the accelerometer watch continuously throughout the 7-day study period and to complete a daily sleep log documenting the times the patch was applied and removed, as well as their bedtime and wake time. Sleep log and actigraphy data were used to identify any lapses or artifacts in the sleep patch data (eg, device malfunctions or failure to apply the device). Participants were instructed to clean their skin with an alcohol prep pad before applying the patch each night at bedtime. They were shown how to apply the patch, gently press it to their skin, start the app on the tablet, toggle the switch to the ON position on the patch, and connect the patch to the app. They were also instructed on how to reconnect the patch to the app if they moved away from the tablet (eg, to use the restroom). Each morning upon waking, participants were shown how to rinse the patch with alcohol spray, set it to dry, and connect it to the magnetic charger to recharge the battery. To increase adherence, a researcher texted participants daily to check in and remind them to apply the patch. Participants returned to the laboratory 3 days later to complete the encoding portion of a memory task (data not presented here). During this second visit, researchers downloaded the data from the tablets and checked it to ensure proper patch functioning and participant compliance. Participants were asked if they had experienced any difficulties using the patch system and were provided with additional instructions if needed. Three days later, participants returned to the laboratory for their third and final visit to complete the retrieval portion of the memory task (data not presented here) and to fill out a debriefing questionnaire with several questions about their experience using the sleep-monitoring system. During the third visit, researchers downloaded all data from the patches before dismantling them to recycle the components.

### Initial Assessment of Data Quality

Before the analysis of sleep stages and microarchitecture, it was important to first ensure sufficient data quality for accurate sleep scoring. First, we computed multitaper spectrograms for each dataset to visualize data quality for an entire night, noting any movements, electrode adhesion issues, or disconnections. Visualizing the spectrograms also allowed us to easily observe, in a single graphic, the frequency power indicative of NREM and REM sleep stages and the repetition of these stages over the course of the night’s recording. To this end, we applied the parameters recommended by Prerau and colleagues [32]. We bandpass-filtered the entire time series for each channel, referenced to the nose, between 0.3 and 35 Hz. We also applied a notch filter to remove any power line noise (59‐61 Hz). Second, data files containing less than 5 hours of recording time were removed from further analysis unless the accelerometer also showed a total sleep duration of less than 5 hours. Given the novelty of this device, we chose to be conservative with the data retained for analysis. We assumed that short-duration data files might have been subject to data loss due to loss of connectivity to the tablet and included these files in this loss category in the Results section. Third, a registered polysomnography technologist, naïve to the source of the data (eg, young, old, estimated sleep duration), visually inspected each data file to make a summary judgment of whether the tracings were of sufficient quality to allow manual scoring without difficulty. Data passing these criteria were included for further analyses described below.

### Sleep Macrostructure and Microstructure Analysis

#### Automated Sleep Stage Scoring

Each night of data for each participant was scored using a previously validated, open-source, automated sleep staging algorithm (Yet Another Sleep Algorithm), which had been previously trained on roughly 30,000 hours of polysomnography data [33]. To reduce processing time and file size, as recommended for this software, all data were first downsampled to 100 Hz. Prior to sleep staging, artifacts were removed from the EEG data using Yet Another Sleep Algorithm’s validated covariance-based automated artifact detection method. EEG data were segmented into 5-second windows, and window-level interchannel covariance metrics were *z* score normalized across the recording. Windows with absolute *z* scores exceeding a threshold of plus or minus 3 were classified as artifacts, ensuring the exclusion of flatlined, noisy, or otherwise problematic data (eg, large movements). Samples corresponding to artifact windows were excluded prior to further analysis. The artifact-cleaned EEG data were then bandpass filtered between 0.1 and 40 Hz. Sleep staging was performed in a fully automated manner in 30-second epochs using a single EEG channel recorded as EEG Ch1-Nose, with an additional EOG bipolar channel (EOG Ch2-EOG Ch1) included to aid in REM sleep detection. The remaining EEG channel (EEG Ch2) was recorded but not used for automated staging. Sleep stage variables (ie, total sleep time, sleep efficiency, and time spent in each sleep stage) were derived for each night and then averaged across all retained nights for each participant to obtain a single per-participant estimate of each sleep variable.

#### Sleep Microarchitecture Analysis

We calculated multiple sleep architecture features using the automated algorithm [33]. SOs were detected at EEG Ch1-Nose. Downsampled EEG epochs were first bandpass filtered between 0.5 and 1.5 Hz. SOs were selected if the negative and positive deflections crossed zero and had a duration between 0.5 and 2 seconds, with an amplitude exceeding the 75th percentile of the dataset’s amplitude. SO density was calculated as the number of detected SOs divided by the minutes of NREM sleep time. We calculated fast spindles separately from 30-second epochs from N2 and N3 sleep stages based on automated staging results. Spindles were detected at EEG Ch1-Nose. The downsampled epochs were bandpass filtered between 12 and 16 Hz. The root mean square was calculated using a sliding window of 0.3 seconds in 0.1-second steps. Spindles were selected if the amplitude of the root mean square exceeded 1.5 SDs for a duration of 0.5 to 2 seconds. Spindle density was calculated as the number of detected spindles divided by the number of minutes spent in each of N2 and N3. NREM relative delta power was calculated from EEG Ch1-Nose to quantify SWA (delta 0.5‐4 Hz) during NREM sleep. EEG data were bandpass filtered between 0.5 and 45 Hz and segmented by N2 and N3 sleep stages. Power spectral density was computed using the Welch method across Delta (0.5‐4 Hz), Theta (4‐8 Hz), Alpha (8‐12 Hz), and Beta (12‐30 Hz) frequency bands. Relative power was calculated as the ratio of power in each band to total power across all bands. A composite NREM relative delta power metric was derived by calculating the duration-weighted average of N2 and N3 relative delta values. Sleep microarchitecture variables were averaged across all datasets available for each participant.

### Ethical Considerations

This study was approved by the University of Texas at Austin Institutional Review Board (STUDY00001995; Biosensing). Informed consent was obtained from all participants prior to their participation. Study data were deidentified, and all personal identifiers are stored in the Health Insurance Portability and Accountability Act (HIPAA)-compliant REDCap database, separate from the study data. Participants were compensated US $150 for their participation. All data were collected prospectively for this project.

## Results

### Participant Experience

We asked study participants to answer several questions about their experiences with our sleep monitoring system. These data are presented in Table 2. As can be seen in the table, young and older adults had similar responses.

**Table 2.** Participants’ experience reports from a debriefing questionnaire^a^.

Question (rating scale)	Young adults, mean rating (SD)	Older adults, mean rating (SD)	*P* value
How comfortable was the sleep mask you wore for seven nights? (1=very uncomfortable; 7=very comfortable)	4.38 (1.36)	5.06 (1.44)	.17
How likely are you to wear this mask again for a different research or clinical study? (1=extremely unlikely; 7=extremely likely)	5.06 (1.61)	5.75 (1.61)	.14
Would you wear this mask if your doctor wanted to determine whether your sleep indicated a sleep disorder? (1=extremely unlikely; 7=extremely likely)	6.53 (0.74)	6.33 (1.59)	.78
How much did the mask interfere with your sleep, if it did? (1=not at all; 7=a great deal)	2.56 (1.5)	1.88 (0.89)	.15
Which option would you prefer: wearing this mask at home for multiple nights or sleeping in a lab while wearing the mask to monitor your sleep? (1=home; 4=no preference; 7=lab)	2.4 (2.23)	1.93 (1.83)	.44
How easy was it to apply the mask? (1=extremely difficult; 7=extremely easy)	4.93 (1.49)	5.73 (1.28)	.12
Overall, how easy was the mask to use? (1=extremely difficult; 7=extremely easy)	5.2 (1.08)	5.5 (1.6)	.26

a Results of Mann-Whitney *U* tests comparing ratings between groups. Statistical significance was set at *P*<.05.

Positive experiences with the system were reported. Specifically, participants noted that the sleep device was easy to use, did not interfere with their sleep, and that they would be likely to wear it again for a research or clinical study. Mann-Whitney *U* test showed that young and older adults did not differ in their ratings of the experience (all *Ps*>.05).

### Data Retention

As can be seen in Table 1, the number of usable nights varied across participants in both age groups. Seven young and 6 older adults provided 0 usable nights of data (Table 1, Panel A). Among these participants, the primary reason for data loss was poor reference electrode adhesion (young: 78%, 38/49; old: 62%, 26/42), with the remaining losses attributable to participant noncompliance, resulting in no data being produced (young: 22%, 11/49; old: 38%, 16/42). Thus, sleep macrostructure and microstructure analyses were conducted on the 11 young and 12 older adults who contributed at least 1 usable night of data. In Table 1, Panel B, “usable nights” is reported as a percentage of nights in which the device was worn, whereas “not worn/connected,” “poor reference electrode adhesion,” and “Bluetooth issues” are each reported as percentages of all scheduled nights. Among participants with greater than or equal to 1 usable night (Table 1, Panel B), the primary source of unusable recordings was poor reference electrode adhesion (young: 48.1%; old: 36.9%). Only a small number of recordings were lost due to inconsistent Bluetooth connectivity. For data that were retained, we computed SNR for each participant separately using power in the delta band before and after sleep onset. As can be seen in Table 1, the average SNR was similar between groups, with little change over the nights of use. These SNR estimates were similar to those obtained in our prior laboratory-based validation study (22.7 dB) [24], suggesting that home use did not diminish signal quality or stability.

### Measurements of Sleep Stages and Microarchitecture

EEG signals can be observed for each of the sleep stages in a representative young and older adult dataset in Figure 3B. Representative spectrograms and associated hypnograms for an entire night from 1 young and 1 older adult are shown in Figure 3C. As is evident in the figure, cycles of sleep stages are visible, with a notable reduction in SWS in the older adult.

Average values for sleep stage and microarchitecture features are shown for each age group in Table 3. Mann-Whitney *U* tests were performed to compare the sleep variables between groups. As can be seen in the table, older adults exhibited lower sleep efficiency (total sleep time divided by time in bed), less time in SWS (N3) and REM, but more time in N2. Regarding the microarchitecture features, spindle activity in both N2 and N3 stages was significantly reduced with age. SWA and SO density were not significantly reduced with age.

**Table 3.** Measurements of sleep stages and microarchitecture averaged across usable nights for each participant, by age group (young adults: n=11; older adults: n=12)^a^.

Measure	Young adults, mean (SD)	Older adults, mean (SD)	Young adults, median (IQR)	Older adults, median (IQR)	Mann-Whitney *U* test	*P* value	Effect size (95% CI)	
TST^b^ (min)	339.95 (31.28)	351.88 (75.95)	351.0 (319.75 to 364.56)	360.2 (328.44 to 393.17)	−18	.27	−0.273 (−0.642 to 0.2)	
Sleep efficiency (%)	82.38 (7.56)	77.22 (11.05)	84.77 (80.38 to 87.04)	79.47 (71.94 to 81.38)	28	.09	0.424 (−0.03 to 0.733)	
Stage 1 (%)	3.23 (1.89)	4.54 (3.38)	2.71 (1.93 to 4.27)	4.65 (1.32 to 6.74)	−11	.50	−0.167 (−0.572 to 0.304)	
Stage 2 (%)	58.60 (10.46)	69.68 (8.76)	55.77 (50.94 to 63.02)	70.25 (64.90 to 75.60)	−39	.02^c^	−0.591 (−0.822 to −0.194)	
SWS^d^ (%)	18.55 (6.7)	10.40 (6.43)	17.59 (14.91 to 22.76)	9.07 (5.52 to 14.23)	42	.01^c^	0.636 (0.263 to 0.844)	
REM^e^ (%)	19.61 (7.55)	15.36 (5.74)	20.90 (14.45 to 26.15)	16.05 (9.82 to 18.38)	27	.10	0.409 (−0.048 to 0.724)	
SO^f^ density	0.79 (0.66)	0.55 (0.31)	0.70 (0.28 to 1.05)	0.58 (0.31 to 0.71)	10	.54	0.152 (−0.318 to 0.562)	
NREM^g^ relative delta power	0.79 (0.05)	0.79 (0.08)	0.81 (0.78 to 0.82)	0.79 (0.77 to 0.85)	4.5	.78	0.068 (−0.392 to 0.501)	
N2 spindle density	2.92 (2.24)	0.94 (1.33)	1.87 (1.48 to 4.64)	0.56 (0.13 to 0.87)	45	.006^c^	0.682 (0.336 to 0.866)	
N3 spindle density	0.25 (0.31)	0.11 (0.15)	0.18 (0.04 to 0.25)	0.06 (0.00 to 0.11)	24	.14	0.364 (−0.101 to 0.698)	

a Results are from Mann-Whitney *U* tests comparing sleep measurements between groups. Effect size is reported as rank-biserial correlation with 95% CIs.

b TST: total sleep time.

c *P*<.05 was considered statistically significant.

d SWS: slow-wave sleep.

e REM: rapid eye movement.

f SO: slow oscillation.

g NREM: non-REM.

## Discussion

### Principal Findings

The goal of this pilot study was to assess the acceptance and feasibility of our newly developed, ultrathin, wearable epidermal electronics system for at-home sleep monitoring in healthy young and older adults. To our knowledge, this is the first report of the home deployment of a forehead, fabric-based electronics, wireless, wearable system for sleep assessment in young and older people. Our results show that (1) participants across young and older age groups rated the system as easy to apply and comfortable to wear during sleep; (2) nights of data were lost for each age group, primarily due to poor adhesion of the reference electrode; (3) signal power was stable over multiple nights of wear at home for retained data; (4) independent (nonauthors) trained sleep technicians verified that they would be able to score retained datasets with little difficulty; and (5) typical age-related differences in both macroarchitecture and microarchitecture features were observed. We discuss these results and their implications below.

Across both young and older adult groups, participants rated the device and tablet application as easy to use, highly comfortable to wear, nonintrusive to their sleep, and something they would use again for both clinical and research purposes. These ratings were as high as or higher than those assessing participant comfort and usability for various wearables, including dry electrode patches for EEG [34], supporting the acceptance of this novel technology for home sleep monitoring in adults across the lifespan. The nose bridge placement for the reference channel is commonly used in many traditional wake EEG studies and in our prior laboratory-based validation study of this sleep monitoring system [24], without similar data loss. Consequently, we used the same electrode placement in this home deployment pilot study. Despite providing thorough instructions to participants—including cleaning the skin with alcohol swabs, washing the patch after every use, and gently pressing the electrodes to the skin—there was a significant amount of data loss, primarily due to poor adhesion of the reference electrode. We believe that a combination of factors, including participants rubbing their nose while sleeping, individual differences in the fit of the electrode over the nose bridge, sweating or oil secretions, and inconsistent adherence to our instructions, contributed to the data loss. In our future work, we will relocate the reference channel to the forehead patch, similar to the location used in other forehead-only EEG systems [16,17], to mitigate these issues.

Finally, results show the age group differences in both macroarchitecture and microarchitecture features typically observed in laboratory-based polysomnography studies [26-29]. Specifically, SWS (N3) was reduced, while N2 was proportionally elevated in older compared to younger adults. Our device was also able to detect NREM microarchitecture features, including SOs and spindles, with spindles particularly attenuated with age. Multiple studies have found age-related attenuation of NREM sleep features, including SWS and spindles, which numerous studies have shown play an important role in the sleep-dependent consolidation of episodic memories [35]. These age-associated reductions are concomitant with age-related reductions in sleep-dependent consolidation, indicated by memory retention, with individual differences in the amount of SWS, as well as the density of spindles, predicting those in memory performance across the adult lifespan [36]. Importantly, low levels of these and other sleep EEG features may predict conversion to mild cognitive impairment or dementia over 3 to 6 years [37] or subtle general cognitive decline in cognitively unimpaired older adults [38]. The present findings highlight the potential for using our sleep patch system to monitor sleep from the comfort of one’s own home to track changes over time in the levels of sleep features that may forecast future cognitive decline.

Our sleep monitoring wearable system is novel in many respects. While wearable sleep EEG monitoring electronic systems exist, such as forehead headbands containing one or several dry electrodes, they are hard and bulky with rigid form factors [13]. They additionally lack electrodes placed to monitor eye movements, which facilitate the scoring of REM sleep. Other wearable systems with smaller form factors, such as those worn around or in the ear, either have to be customized for an individual for optimal fit [39] or contain wires [21,39], which could contribute to relatively low signal quality due to mechanical noise [40]. Our wearable system, with its wireless nanomembrane electrodes embedded in soft adhesive fabric, is designed to maximize comfort, ease of application, and obtain reliable signals over multiple nights. The Bluetooth-based wireless data transmission to a mobile tablet with a simple user interface offers additional portability and accessibility for our system. Finally, the more costly circuit components can be recycled, reducing production costs and negative environmental impacts. As discussed in our prior work, our manufacturing process is highly scalable, making it possible to produce and ship devices to a large number of participants.

### Limitations

There are a few limitations to note. A primary limitation of the current home-deployment prototype relates to data loss, which occurred mostly due to the instability of the reference electrode placed on the bridge of the nose. While this placement was effective and not problematic in our prior laboratory-based validation study, home-based recordings introduced additional variability due to interindividual differences in facial anatomy and sleep-related movements. In particular, contact with bedding during sleep and variations in nasal shape made stable adhesion difficult for some participants, resulting in unusable recordings when adhesion was insufficient. These findings highlight a limitation specific to unsupervised home use that could not have been fully anticipated from laboratory testing alone. To address this issue, we are developing updated designs in which all electrodes, including the reference, are integrated into a single forehead patch, relocating the reference electrode to a mechanically more stable position to improve robustness and data retention during future home deployments. Given that greater attenuation of certain sleep features, including SWS, may predict later cognitive decline, it would be of great interest to identify individuals at the greatest risk of decline. Future studies recruiting larger samples of participants and tracking their sleep and cognitive changes over time would allow for such investigations.

### Conclusion

Our system overcomes the limitations of home polysomnography and wearable sleep monitoring devices with regard to comfort, sustained signal reliability, and accurate sleep analysis. These results help lay the groundwork for studies in participants at risk for AD to determine whether particular sleep EEG patterns, measured accurately from one’s own home, can help identify individuals most likely to exhibit cognitive decline. Hesitancy to participate in research is well known among historically minoritized individuals (eg, [41]), and furthermore, individuals with poor sleep may be hesitant to participate in a laboratory-based sleep research study. Our sleep monitoring patch, which is manufactured using laser micromachining and printing, is highly scalable and could be shipped to large numbers of participants for future cognitive and clinical studies. Sleep architecture results could potentially differ as a function of age and/or race or ethnicity and could serve as targets for future personalized brain stimulation, cognitive behavioral therapy, or pharmacological interventions.

## Abbreviations

AD: Alzheimer disease
EEG: electroencephalography
EMG: electromyography
EOG: electrooculography
HIPAA: Health Insurance Portability and Accountability Act
NREM: non-REM
REDCap: Research Electronic Data Capture
REM: rapid eye movement
SNR: signal-to-noise ratio
SO: slow oscillation
SWA: slow-wave activity
SWS: slow-wave sleep
